# Testing the Purity of *Limnospira fusiformis* Cultures After Axenicity Treatments

**DOI:** 10.3390/cells14020136

**Published:** 2025-01-17

**Authors:** Michael Schagerl, Alexander Kaptejna, Fabian Polz, Sameh S. Ali, Shuhao Huo, Joana Seneca, Petra Pjevac, Vera Hechtl

**Affiliations:** 1 Department of Functional and Evolutionary Ecology, University of Vienna, Djerassiplatz 1, A-1030 Vienna, Austria; michael.schagerl@univie.ac.at (M.S.); a01040257@unet.univie.ac.at (A.K.); a11724683@unet.univie.ac.at (F.P.); 2 Botany Department, Faculty of Science, Tanta University, Tanta 31527, Egypt; samh_samir@science.tanta.edu.eg; 3 School of Food and Biological Engineering, Jiangsu University, Zhenjiang 212013, China; huo@ujs.edu.cn; 4 Joint Microbiome Facility of the Medical University of Vienna and the University of Vienna, Djerassiplatz 1, A-1030 Vienna, Austria; joana.silva@univie.ac.at (J.S.); petra.pjevac@univie.ac.at (P.P.); 5 Department of Microbiology and Ecosystem Science, Centre for Microbiology and Environmental Systems Science, University of Vienna, Djerassiplatz 1, A-1030 Vienna, Austria

**Keywords:** algal culture, bacterial contamination, sterile, cultivation, plate test

## Abstract

Contaminations are challenging for monocultures, as they impact the culture conditions and thus influence the growth of the target organism and the overall biomass composition. In phycology, axenic cultures comprising a single living species are commonly strived for both basic research and industrial applications, because contaminants reduce significance for analytic purposes and interfere with the safety and quality of commercial products. We aimed to establish axenic cultures of *Limnospira fusiformis*, known as the food additive “Spirulina”. Axenicity is strived because it ensures that pathogens or harmful microorganisms are absent and that the harvested biomass is consistent in terms of quality and composition. For the axenic treatment, we applied sterile filtration, ultrasonication, pH treatment, repeated centrifugation, and administration of antibiotics. For testing axenicity, we considered the most common verification method plate tests with Lysogeny Broth (LB) medium, which indicated axenicity after treatments were performed. In addition, we included plate tests with Reasoner’s 2A (R2A) agar and modified Zarrouk+ medium, the latter comparable to the biochemical properties of *L. fusiformis*’ cultivation medium. In contrast to LB plates, the other media, particularly Zarrouk+, indicated bacterial contamination. We conclude that LB-agar plates are inappropriate for contamination screening of extremophiles. Contamination was also verified by cultivation-independent methods like flow cytometry and 16S rRNA genome amplicon sequencing. We detected taxa of the phyla Proteobacteria, Bacteriodota, Firmicutes and to a lesser extent Verrucomicrobiota. Contaminants are robust taxa, as they survived aggressive treatments. Sequencing data suggest that some of them are promising candidates for in-depth studies to commercially exploit them.

## 1. Introduction

Axenic strains are cultures of a single species, free of other living organisms. The term “axenic” (Greek = free from foreign life) was introduced by Baker and colleagues in 1942 and recently extended to virus particles by some authors, e.g., Vu et al. [1]. When performing molecular, biochemical, or physiological experiments such as identifying biological producers of bioactive compounds, axenic states are desirable to eliminate immeasurable effects that could interfere with the experimental result [2,3]. Also, for genetic engineering, axenicity is recommended [4]. True axenicity is desirable for certain research questions, otherwise antagonistic, commensalistic, and competitive relationships cannot be ruled out [1]. Application examples are studies of host–microbe interactions investigating single-celled hosts [5,6], but also multicellular organisms such as insects [7,8]. For biotechnological and industrial applications, axenicity is also desirable [1]. It improves product quality, helps to minimize the risk of unwanted side products and the production of toxins, which is of special importance for pharmaceutical products, food and dietary supplements. Nevertheless, contaminations can hardly be avoided especially when conducting long-term experiments or large-scale cultivation. For industrial cultivation, providing sterile medium and sterilization of large photobioreactor volumes pose limitations for axenicity. Large-scale cultivation is like a race between target organisms and contaminants with amoeba, rotifers, and ciliates often creating serious problems [9,10].

Methods to isolate unialgal cultures from bacterial associations regardless of their mutualistic or antagonistic relation include manual isolation of single algal cells by means of capillary pipetting, fluorescence-activated cell sorting, plate spreading, combinations of centrifugation cycles, sterile filtrations, serial dilutions, pH treatments, and antibiotic treatments [2], with sterile laboratory conditions highly recommended for all working steps. The first two techniques focus specifically on the cells of interest, the other methods are based on trial and error, because the specific strains are not selected before cultivation. Commonly, physical and chemical treatments are combined to eliminate the contaminants, as one single approach does not suffice. Treatments must be adapted to the target organism, as the preferred living conditions and resistance against adverse conditions can vary in extremes. The biogeochemical properties of the natural environment from where the target organism was collected may serve as first assessment for preferred culture conditions (e.g., salinity, temperature, alkalinity, irradiation, and nutrient supply). For maintaining axenic conditions, special care must be taken for inoculation of the culture into a new medium.

One central issue is to verify axenicity. Several research works have been published with ‘axenic’ cultures, but ‘non-presence’ cannot be assessed [11]. The probability of true axenicity can, however, be increased by using modern screening methods such as flow cytometry (FCM) or sequencing [3], at the best used in combination. Pokorny et al. [11] proved 16S rRNA gene amplicon sequencing among other methods (epifluorescence microscopy, FCM) as the method of choice to identify microbial contaminants in *Limnospira fusiformis* cultures. Axenic strains can be ordered from culture collections, but culture collections often use agar plates for axenicity tests, which have a very limited informative value [11], so verifying axenicity before running experiments is paramount [12]. Some studies claimed to have developed methods to achieve axenicity, but they also used plate tests of only limited value (e.g., [13,14,15,16]). In other methodological studies, the screening period after axenicity treatment was very short [2,4,17]. In particular for dormant stages of contaminants and slowly growing strains, elongated periods under review are highly recommended.

Axenic cyanobacterial cultures have been isolated for various taxa, such as *Aphanizomenon* and *Oscillatoria* [14], *Phormidium* [18] and also *Limnospira* [11,19,20]. Pros and cons of axenic cultures in cyanobacterial studies were comprehensively reviewed by Dextro et al. [21]. The methods to achieve axenic cultures for *Limnospira* included both physical and chemical treatments, with use of antibiotics. Choi et al. [20] used a two-step procedure with repeated rinsing of the filaments, followed by a cocktail of antibiotics. Pokorny et al. [11] followed the protocol of Sena et al. [19] with a combination of physical and chemical treatments, although with some modifications: the treatment included filtration steps, pH treatment, antibiotics, and serial dilution. For the antibiotic treatment, three different approaches were chosen: a standard β-lactam antibiotic treatment according to Sena et al. [19], an ultrasonication step before adding the antibiotics to remove attached bacteria, and an additional antibiotic (chloramphenicol) added to the standard β-lactam antibiotic mix. β-lactam antibiotics target the catalytic activity of transpeptidases involved in establishing peptide bonds necessary for peptidoglycan synthesis as part of a bacterial cell wall structure and are most efficient in Gram-positive bacteria [22]. *L. fusiformis* as a Gram-negative bacterium is not as much affected by β-lactam antibiotics as Gram-positive bacteria. Chloramphenicol inhibits cellular protein synthesis; therefore, addition to the mix of β-lactam antibiotics is assumed to increase efficacy of the antibiotic treatment. Moreover, glucose as a nutrient source and growth initiator of heterotrophic bacteria was added to minimize dormancy states and thus further increase antibiotic efficacy, which mostly targets active populations. This treatment is based on the observations of Levin-Reismann et al. [23], who investigated antibiotic tolerance in *Escherichia coli*. It turned out that dormant or slowly growing sub-populations proliferate after the antibiotic treatment was terminated. According to Choi et al. [20], their axenic treatments were assumed to be successful after absence of evidence of bacteria in plate tests for 2 days. Sena et al. [19] performed visual inspections directly after treatments, and plate tests with standard broth, without providing any further details. Pokorny et al. [11] applied sophisticated methods to assess axenicity. Considering these modern methods, bacterial contamination, however, became evident even after serious axenicity treatments, which went beyond that of Sena et al. [19]. With only applying methods used by former *Limnospira* studies, the axenicity treatments would have been assumed to be successful.

**Figure 1:**
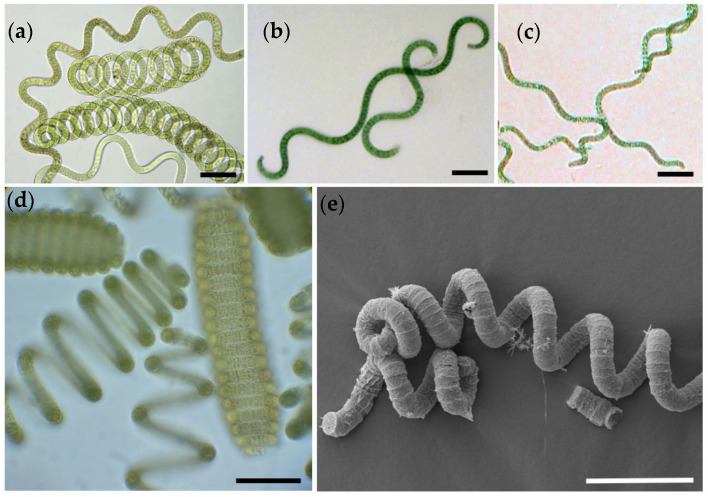
*Limnospira fusiformis* clones used in this study. (**a**) Nakuru; (**b**) Big Momella; (**c**) Arenguade; (**d**) natural sample of *Limnospira* showing the characteristic coils of the filaments; (**e**) SEM microphotograph of *Limnospira fusiformis* clone Nakuru after critical point drying. Scale bars are 50 µm for (**a**–**d**), and 20 µm for (**e**).

In this study, we focused on the cyanoprokaryote *Limnospira fusiformis*, formerly known as *Spirulina platensis* and *Arthrospira fusiformis* [24]. Nowadays, the genus *Spirulina* is placed in the order Spirulinales, as it differs from *Limnospira* both phylogenetically and cytologically [25]. *Limnospira fusiformis* is placed in the order of Oscillatoriales. Cells are arranged in unbranched trichomes with visible cross walls (Figure 1). The trichomes are mostly coiled; filaments have a diameter of approximately 7 to 10 µm, the spirals 20 to 50 µm. *Limnospira,* however, changes its morphology from densely coiled filaments to wavy formed trichomes to completely straight forms [26,27]. Natural habitats are East African soda lakes, where it thrives under high pH, high salinity, and high turbidity [28]. The cyanobacterium is rich in proteins, minerals, essential fatty acids, carotene, and vitamin B12 analogous, and has a wide range of applications, including as feed for animal farming and aquaculture, and in recent years, as food additive for humans [20].

We aimed to improve the axenic treatment as developed by Pokorny et al. [11] with a focus on eliminating bacterial contamination, as the cultures of interest are already free of eukaryotic contaminants and other cyanoprokaryotes. We assume that by enhancing the concentration of chloramphenicol, contaminants will be eliminated. We used modern methods to prove axenicity, but also included simple plate tests with both standard media Lysogeny Broth (LB) medium, Reasoner’s 2A (R2A) agar and a medium comparable to Zarrouk used to grow *Limnospira*. We propose that agar plate media with biochemical properties similar to the original algal cultivation medium will be more reliable in the verification of bacterial contaminants compared to the commonly used LB and R2A media. 16S rRNA gene amplicon sequencing was used to characterize the bacterial community in the original cultures and to assess axenicity.

## 2. Materials and Methods

This study was conducted with clonal *Limnospira fusiformis* strains (Algensammlung Wien, algae culture collection of the University of Vienna, Austria, ASW 01 100 ‘Nakuru’, ASW 01 101 ‘Big Momella’, ASW 01 102 ‘Arenguade’; Figure 1). Cultures are maintained in Zarrouk cultivation medium [29] at 25 °C with a light:dark cycle of 12:12 and an intensity of 25 µmol photons m^−2^ s^−1^ (warm-white fluorescence tubes). All steps were performed under sterile conditions in a ventilated safety cabinet (SafeFAST Classic 212, Class II Biological Safety Cabinet, Milano, Italy); glassware and tools were autoclaved.

Before axenic treatments took place, pre-cultures were grown for several weeks with repeated inoculations in biweekly intervals to guarantee vital, healthy filaments. Optical density as a proxy for biomass was measured repeatedly at 750 nm to monitor culture development (U-2000 photometer, Hitachi, Tokyo, Japan); optical density of the pre-cultures was between approximately 0.02 (just after inoculation) and 1.00 just before inoculation. All treatments were conducted at 25 °C with a day:night cycle of 16:8 and 50 µmol photons m^−2^ s^−1^. This setting was also kept for cultivation after treatments.

**Figure 2:**
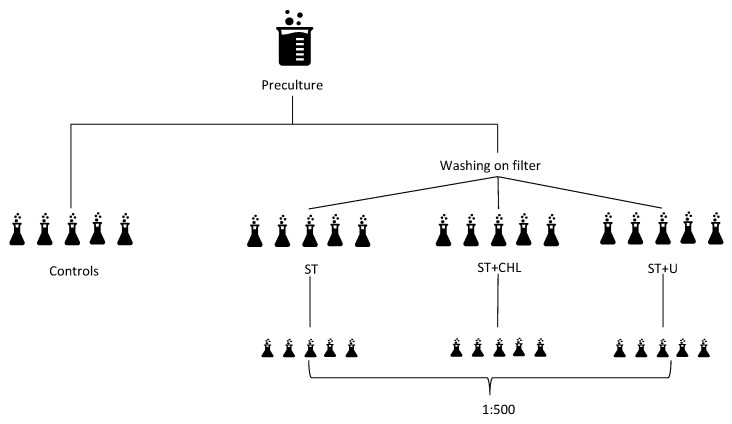
Steps and approaches to reach axenicity. ST = standard treatment, ST + CHL = standard treatment + chloramphenicol, ST + U = standard treatment + ultrasonication, and 1:500 = dilution step. Overall treatment lasted for five days (see Table 1 for details), followed by four weeks growth with repeated testing for axenicity.

Working steps of the axenic treatments are shown in Figure 2. Treatment started with rinsing the filaments on membrane filters with bicarbonate-free Zarrouk medium (Isopore hydrophilic polycarbonate, 3 µm pore size, Merck KGaA, Darmstadt, Germany) to get rid of most contaminants in the liquid medium. The filaments were then transferred into Erlenmeyer flasks and split into three groups (Figure 2), each group containing five replicates. The groups were then subjected to different axenic treatments (Table 1): (1) standard treatment (ST), (2) ST + chloramphenicol and glucose (ST + CHL), and (3) ST with ultrasonication (ST + U). ST followed the protocol of Sena et al. [19]: the pH was raised to 12 by adding 1 M NaOH drop by drop and the samples were then kept for 72 h at pH 12 to exacerbate living conditions for unwanted heterotrophic bacteria. To replace the alkaline medium with full Zarrouk medium, three centrifugation cycles of 10 min each at 3000 rcf and 20 °C were performed. After each cycle, the medium was replaced, and the pellet dispersed by vortexing. After this step, one group was treated with an additional ultrasonication step (ST + U) to remove bacteria attached to the filament surface of *Limnospira*. Samples were subjected to 12 × 10 s rounds of ultrasonication (Sonifier 250, Branson, Danbury, Connecticut) at the lowest intensity with ice water cooling intervals of 30 s between rounds. The detached mucus was removed by 3 × 10 min centrifugation cycles as previously described. All samples were then treated with four different antibiotics: ampicillin (61.6 µg mL^−1^), penicillin (85.8 µg mL^−1^), cefoxitin (76.9 µg mL^−1^) and meropenem (38.9 µg mL^−1^). For the group with chloramphenicol addition (ST + CHL), 10.0 µg mL^−1^ chloramphenicol was added as well as 100 µg mL^−1^ glucose. After 48 h of cultivation in the dark, three centrifugation cycles were performed as mentioned above to remove the antibiotics from the medium, followed by a 1:500 dilution to enhance the chance of bacteria-free cultures. Untreated control samples were included for comparison with axenic treatments.

### 2.1. Harvest, Sample Fixation, and Storage

The controls were harvested at an OD of approximately 1.0 by filtering 8 mL suspended culture on autoclaved filters (Isopore hydrophilic polycarbonate, 3 µm pore size, Merck KGaA, Darmstadt, Germany). For FCM analysis, 588 µL of the filtrate and 12 µL glutardialdehyde were added to cryogenic storage vials, which were then shock-frosted in liquid nitrogen for 30 min and stored at −80 °C until analysis. Cultures with axenic treatments were harvested after 3 days, 1 week, 2 weeks, and 4 weeks, respectively, in the same way as described above. The repeated harvests over longer periods were performed to consider both fast and slowly growing contaminants.

### 2.2. Plate Tests

To test the presence of heterotrophic contaminants, quintuplicates of agar plates (agar 1.5%), prepared with three media LB medium (Miller formulation with 10 g L^−1^ NaCl; VWR International J106, Vienna, Austria), R2A medium (VWR International 100416, Vienna, Austria), Zarrouk+ medium with organic ingredients added (glucose 1.5%, peptone 0.5% + yeast extract 0.3%), were inoculated with 50 µL sample each. The agar plates were then stored at 25 °C in the dark and screened after 3 days, 1 week, 2 weeks, and 4 weeks of inoculation.

### 2.3. Flow Cytometry Analysis

To quantify heterotrophic contaminants, a flow cytometer (Amnis CellStream, New York City, NY, USA) equipped with a 488 nm blue light laser was used. Samples were thawed and stained with SYBR Green I nucleic acid stain (Invitrogen Thermo Fisher Scientific Inc., Waltham, MA, USA) at a volume ratio of 1:10,000. Incubations took place at 37 °C for 13 min in the dark. Four measurements for event counts, 30 s were taken for each sample. A gated area was chosen by counting the in-size relevant events of the two parameters 488–611/31-C5 versus 488–528/46-C3. Sample dilution (Milli-Q water) was only necessary for controls (1:60), ST + CHL samples after week 1 until the end of the monitoring (1:10), and for the other treated samples from week 2 and week 4 after inoculation (1:10).

### 2.4. 16S rRNA Gene Amplicon Sequencing

DNA extractions using the DNeasy PowerSoil Pro Kit (QIAGEN, Venlo, The Netherlands.) and Illumina MiSeq-based highly multiplexed 16S rRNA gene amplicon sequencing (Illumina Inc., San Diego, CA, USA) were performed by the Joint Microbiome Facility of the Medical University of Vienna and the University of Vienna (JMF). For the 16S rRNA gene amplification, the following oligonucleotide primers were used to amplify the V4 regions of the 16S rRNA gene fragments: 515F (GTGYCAGCMGCCGCGGTAA), 806R (GGACTACNVGGGTWTCTAAT) [30,31]. For the unique dual barcoding approach (UDB-H12), a unique 12 nt barcode sequence (5′-bc12_1-H1-3′) and unique bc12-H2 fusion primer (5′-bc12_2-H2-3′) were used. The standard operating procedures (SOPs) for amplicon sequence generation and analysis performed at the JMF were carried out as described by Pjevac et al. [32]. Amplicon sequence variants (ASVs) were determined using the DADA2 R package Version 4.3.0, following the workflow detailed in [33,34]. FASTQ reads 1 and 2 were trimmed at 220 nt and 150 nt, respectively, with an allowance for expected errors of 2 and subsequent classification of ASV sequences was performed using SINA version 1.6.1 and the SILVA SSU Ref NR 99 database, release 138.1, using default parameters [35,36]. The data have been submitted to NCBI’s Sequence Read Archive (SRA) under BioProject accession number PRJNA866304.

### 2.5. Statistics

IBM SPSS Statistics (version 28.0.0.0) was used to perform rm-ANOVAs and Bonferroni post hoc tests on the FCM data, using cells mL^−1^ of controls and treated samples to check for significant differences between harvests and treatments.

## 3. Results

Since the same pattern was observed in all three strains, we focus here on the results of a single clone ‘Nakuru’. Overall, the three axenic treatments showed a significant reduction in heterotrophic contaminants compared to the control (rm-ANOVA, Bonferroni post hoc test *p* < 0.001). Detection of contaminations was dependent on the screening method applied. With only results of LB-agar plates considered, axenicity would have been achieved (only seemingly). After taking all results together, bacterial contamination was detected in all treatments. After four weeks of cultivation, filament growth was still not visible to the naked eye. Nevertheless, in the following month, all treated clones densified to characteristic dark blue-green cultures, containing vital, healthy filaments of *Limnospira*.

### 3.1. Agar-Plate Tests

**Figure 3:**
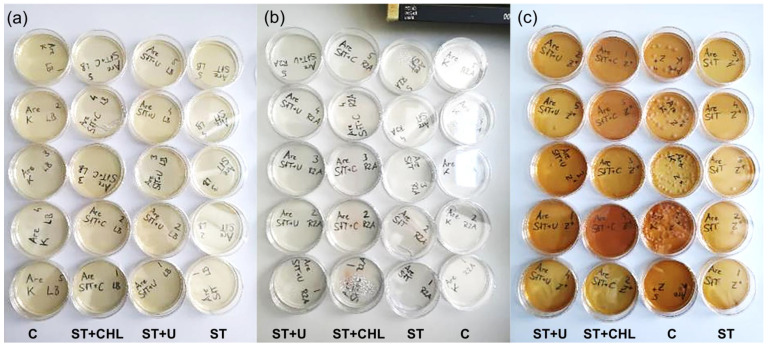
Plates 3 days after inoculation: (**a**) No colonies are visible for any of the treatments on LB medium. (**b**) In R2A medium, a few bacterial colonies were monitored for all treatments with highest contaminations in controls (red clusters). (**c**) Contamination was confirmed across all treatments on agar plates with Zarrouk+ medium. Abbreviations indicate steps to reach axenicity: C = control without dilution, ST = standard treatment, ST + CHL = standard treatment + chloramphenicol, and ST + U = standard treatment + ultrasonication.

LB medium plates did not show any bacterial growth after treatments, not even after 4 weeks. Controls on R2A and Zarrouk+ medium had subtle agglomerations of red colonies 3 days after inoculation (Figure 3, Table 2). ST + CHL-treated cultures on Zarrouk+ and R2A medium had similar bacterial lawns (Figure 3) with treated samples showing fewer colonies of bacteria.

**Figure 4:**
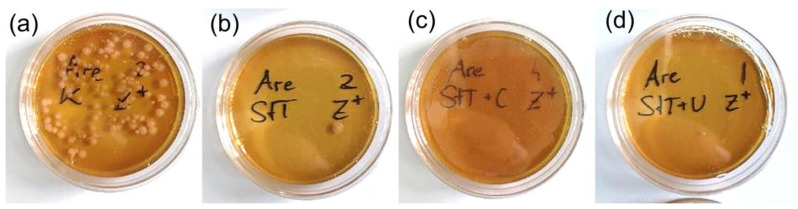
Zarrouk+ medium after 3 days inoculation: (**a**) control, (**b**) standard treatment (ST): single distinct colony, (**c**) ST + chloramphenicol (ST + C): “nebula” of colonies, and (**d**) ST + ultrasonication (ST + U): single tiny colonies.

The highest growth of contaminants by far occurred on Zarrouk+ medium plates with controls (Figure 3 and Figure 4). At 1 week after inoculation, colonies of heterotrophs grew in size and the white lawn was accompanied by a red/pink lawn (Table 2). After some time, additional colonies started to form.

On Zarrouk+ plates, new colonies were visible on ST + CHL plates and snowflake-like expanding colonies on ST + U plates 2 weeks after the experiment started. Contrarily, hardly any visible changes could be recognized on R2A and LB medium. At 4 weeks after inoculation, no further development was observed.

### 3.2. FCM Analysis

**Figure 5:**
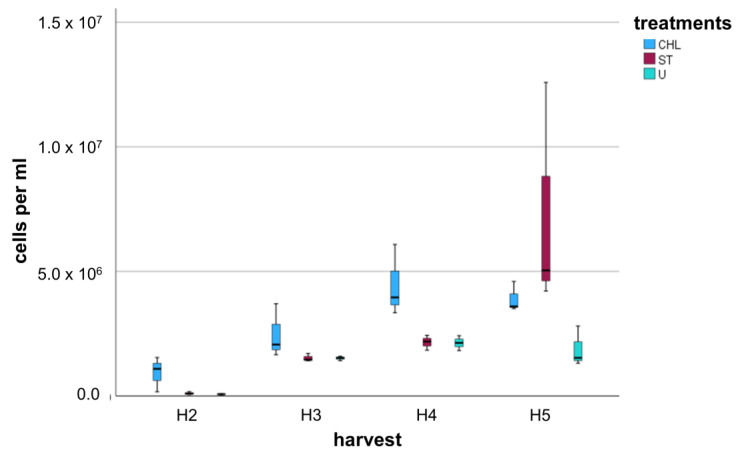
Boxplot of FCM cell counts of treated cultures for each harvest (H2 = 3 days, H3 = 1 week, H4 = 2 weeks, H5 = 4 weeks after completion of treatment, n = 3). CHL = standard treatment + chloramphenicol, ST = standard treatment, and U = standard treatment + ultrasonication. For better visibility, controls containing approximately 4.5 E5 cells mL^−1^ were excluded.

The event count of the gated area (cell count mL^−1^) was compared for the controls and each treatment per harvesting time (Figure 5). The cell count of the controls was approximately 4.5 × 10^7^ cells mL^−1^ and significantly higher compared to all treatments. We found a significant difference in cell counts 4 weeks after treatments compared to the previous harvests (rm-ANOVA, Bonferroni post hoc test *p* < 0.001). Noticeable is also the stagnation of cell number for ST + CHL and ST + U between the last two harvests.

### 3.3. 16S rRNA Gene Amplicon Sequencing

**Figure 6:**
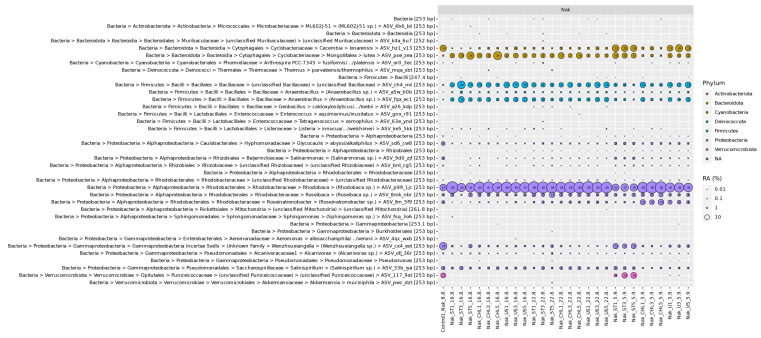
Sample names include the different replicates that were used together with the date on which they were sampled. 16.8. is H2, 22.8. is H3 and 5.9. is H4. Bubbles indicate the contamination found in each sample with different sizes and colors corresponding to the fraction of reads per library RA (%) and the phylum each amplicon sequence variant (ASV) was assigned to, respectively. Fractions displayed for higher taxonomic ranks exclude those for separately shown lower taxonomic ranks.

To further identify the contaminants, a 16S rRNA gene amplicon sequencing analysis was performed (Figure 6). Some sequences indicate the preference of the contaminants towards saline-alkaline conditions, e.g., *Rhodobaca* sp., *Salinispirillum* sp. and *Wenzhouxiangella* sp. By far the highest relative abundance of 16S rRNA gene ASVs across all samples belong to the phylum Proteobacteria, more specifically the genus *Rhodobaca*, followed by the phyla Bacteriodota and Firmicutes. Also, some growth patterns can be deduced (Figure 6). *Rhodobaca* sp. was growing very fast compared to other taxa, but at the final harvest, their relative number already decreased for the ST and ST + U treatments. Other taxa, such as *Roseinatronobacter* sp., *Wenzhouxiangella* sp. (Proteobacteria), and *Cecembia lonarensis* (Bacteriodota), developed slower. Bacteriodota were sensitive against ST + CHL treatment, some Proteobacteria such as *Wenzhouxiangella* sp. and *Salinispirillum* sp. and unclassified Verrucomicrobiota were also sensitive against ST + U application.

## 4. Discussion

None of the cultures turned out to be axenic after treatments. R2A and Zarrouk+ plates showed the cell growth of contaminants already after a few days of inoculation, but not LB plates, which are obviously inappropriate to prove axenicity for extremophilic phototrophs. This result confirms the study conducted by Pokorny et al. [11], where LB-agar plates did not show any contaminants and would have inferred apparent axenicity. This phenomenon is explained by the “great plate anomaly”, already formulated by Staley [37], which suggests that most microbes are hard to cultivate. We conclude that LB-agar is an unsuitable method to test for axenicity, at least for cultures of extremophilic species such as *L. fusiformis*. Heterotrophic contaminants in the original pre-culture samples are adapted to the biochemical properties in the Zarrouk medium and, therefore, flourished on the Zarrouk+ plate medium. The LB-agar used in the current study contained 10 g L^−1^ NaCl and is adjusted to pH = 7, which is an unfavorable environment for alkaliphilic organisms.

The presence of alkaliphilic contaminants was confirmed by the sequence data. We detected *Rhodobaca*, *Roseinatronobacter*, *Wenzhouxiangella*, *Cecembia lonarensis*, and *Mongoliitalea lutea*, the latter two belonging to Cyclobacteriaceae (Bacteroidota), amongst other heterotrophs (Figure 6). It should be mentioned here that the genus *Rhodobaca* was initially described from the saline-alkaline Lake Bogoria (Kenya) as *R. bogoriensis* [38], which is located in the East African Rift Valley and one of the natural habitats of *Limnospira fusiformis* [39]. Alkaliphilic bacteria of the family Rhodobacteraceae (*Rhodobaca*, *Roseibaca*, *Roseinatronobacter*), maybe also *Cecembia lonarensis* and *Mongoliitalea lutea* (Cyclobacteriaceae), were likely responsible for the reddish colonies observed in the plate tests. Although contaminants are not the desired target in a study focusing on axenic treatment, this result encourages out-of-the-box thinking. Alkaliphilic bacteria, their enzymes and metabolites turned out as promising for various industrial and biotechnological applications [40,41,42,43]. Rhodobacteraceae and Cyclobacteriaceae, as also detected in our cultures, have high capabilities to decompose landfill leachate [44]. The compatible solute ectoine, industrially applied to stabilize cells and macromolecules, was detected in Rhodobacteraceae [45]. Their reddish color is attributed to carotenoids, which are promising for biotechnological exploitation.

Choi et al. [20] stated that plate tests are rather time intensive and should be examined two weeks after inoculation. Nevertheless, some slow-growing prokaryotes might take up to 50 days to form visible colonies to the naked eye, under the pre-condition of their proper growth conditions [1]. In our case, these concerns are irrelevant, as colonies were already visible only three days after inoculation on the appropriate Zarrouk+ medium and, therefore, proved non-axenicity. However, 16S rRNA gene amplicon data obtained in the current study point to large differences in growth rates, with some taxa becoming relatively more abundant after 4 weeks of inoculation (Figure 6).

A promising method to quantify bacterial numbers is FCM. Although the device is quite expensive, analyzing samples is low cost, rapid, and the method is reliable to measure individual particle volume, light scatter, and fluorescent properties [46,47]. Compared to traditional cell counts by means of microscopy, sample size and counted particle number are much higher with FCM, which increases the statistical power. Sample throughput is considerably higher compared to microscopy, and the result does not depend on the experience of the laboratory technician. However, FCM has also to handle with difficulties. Amongst these are large particles, which may clog the nozzle. Complex particle shapes are another challenge for counting. Sometimes particles have the tendency to clump together, which is also challenging for FCM counts. This can be caused by the shape itself, e.g., the twisted filaments of *Limnospira* or by excreted substances, such as sticky mucus of extracellular polymeric substances (EPS). Interpretation of obtained FCM data must be performed with care, because a differentiation between living and dead contaminants is not possible with standard dyes, such as the fluorescent stain SYBR Green I used in the current study. As a membrane-permeable dye, SYBR Green I unselectively stains DNA of living and dead organisms. Selective staining methods for living organisms are available, such as fluoresceindiacetate or SYTO 9 combined with propidium iodide, but they also have drawbacks and are not easy to perform.

FCM analysis indicated contaminants in all treated samples and controls. Cell numbers were, as expected, highest in controls, exceeding the treated cultures by orders of magnitude. ST samples showed a consistent increase in cell counts until four weeks after treatments. ST + CHL-treated samples were higher in total contaminant cell numbers than ST and ST + U-treated samples. However, cell propagation visibly reached the stationary phase after four weeks of inoculation. This also applied to ST + U-treated samples, although their initial cell number was in the range of ST-treated samples. Even though we applied a higher concentration of 10.0 µg mL^−1^ chloramphenicol compared to the study of Pokorny et al. [11] (6.8 µg mL^−1^), ST + CHL-treated samples had the highest cell number after the first harvest compared to the other treatments, which suggests that adding chloramphenicol and glucose to the β-lactam antibiotic mix is not appropriate to reduce the overall number of contaminants. The impact of adding glucose to terminate dormant states of bacteria and to lower tolerance against antibiotics remains unclear.

Concerning the physical control techniques applied, we decided on a combination of filtration [48], dilution [49], centrifugation [50], and ultrasonication [49]. Fluorescent-activated cell sorting is a very promising and efficient way of isolating single cells, but is not suitable for filamentous cyanobacteria like *Limnospira*, *Nostoc* or *Anabaena*. The chemical control techniques were antibiotic treatments [18,19,20,51], and a treatment at a pH of 12 [19].

An efficient method to check for axenicity is 16S rRNA gene amplicon sequencing [11]. The two most abundant families in this experiment were Rhodobacteraceae and Cyclobacteriaceae, which appear to be the most persistent contaminations. It might be useful to keep this in mind in future axenicity treatments for fighting contaminants. Nevertheless, it is important to consider that 16S rRNA gene analysis detects DNA from both living and dead cells, potentially leading to false-positive results [52]. We, therefore, recommend repeated sequencing over four weeks to verify changes in microbial community structure.

As none of the antibiotic treatments turned out to be successful, it remains unclear how true axenicity in algae cultures can be achieved. Authors who claim to have developed methods, e.g., by treatment combinations, lack appropriate axenicity verification methods. Choi et al. [20] applied antibiotics treatment by consecutively applying imipenem, neomycin, and cycloheximide. The authors used R2A agar, which is common for testing drinking water. R2A medium was developed for slowly growing bacteria that will not readily stablish on nutrient-rich media, and therefore does not seem to be appropriate for extremophiles testing. Choi et al. [20] also referred to their cultures as axenic only two days after inoculation on several media, which is too short. Doppler et al. [2] only used LB-agar plates to verify axenicity after four weeks, which turned out to be an inappropriate medium for the *Limnospira* axenicity tests in our study. We suggest combined, repeated FCM analysis and 16S rRNA gene amplicon sequencing to enhance the probability of detecting contaminants, or in other words to assume axenicity if no contaminants are detectable. In a survey of several algae culture collections, Pokorny et al. [11] mentioned that only 1 out of 13 culture collections that maintain axenic cultures test their cultures regularly using FCM.

The question arises if true axenicity is mandatory (see also [21]). It is plausible if biomass is generated for food production or pharmaceutical applications to minimize the risk of pathogens or toxic by-products and to guarantee consistent quality of the products. For many application fields, unialgal cultures, however, seem to be sufficient. Additional bacterial communities in the cultures might even be essential for growth-enhancing effects of mutualistic bacteria–microalgae associations, because metabolic waste is decomposed by heterotrophs, and nutrient supply enhanced in the direct surrounding of the phototrophs [53,54,55]. For certain applications, axenic cultivation is not the primary target, it is rather to generate well performing cultures, for which also algae–bacteria interactions may be advantageous [56]. Phytoplankton–bacteria communities represent the most important association in aquatic environments, as phytoplankton are the dominant primary producers at the base of food webs [57,58]. Marine heterotrophic bacteria satisfy their carbon demand of up to 50% by consumption of phytoplankton. In reverse, if the supply of allochthonous macronutrients is low, phytoplankton benefits from remineralized nutrients from bacteria, such as nitrogen, phosphorous, and some vitamins (e.g., B12) [59]. *Limnospira fusiformis* is an alkaliphilic species thriving in saline inland waters, even at salinity beyond that of seawater. For freshwater *Limnospira*, no data exist to our knowledge. Bacteria are also attached to the mucus of EPS secreted by microalgae during growth [1,21]. Although little study has yet been devoted to bacterial–algal interactions [54], many microalgae species thrive only with bacterial symbionts [53,55], which could also enhance the production of valuable compounds for industrial or environmental purposes [1,21,54,60]. As such, the role of the phycosphere, the immediate surrounding of the algal cell enriched in organic nutrients, remains unclear [21,58].

The question remains if other studies really resulted in true axenicity, or if axenicity was assumed just because of inadequate testing methods, because “absence” is hard to verify. Each of the screening methods has advantages, but also drawbacks: 16S rRNA gene amplicon sequencing and FCM for example cannot distinguish between free living or dead cells and plate tests need to be carefully evaluated to account for plate anomalies. Therefore, combining different methods is crucial to enhance the probability of true axenicity and to ensure reliable experimental outcomes. As previously mentioned, the most promising approach is repeated, combined FCM and 16S rRNA gen amplicon sequencing. An inexpensive alternative is plate tests with a medium with similar biochemical properties to the original medium, producing visible and reliable results already after three days of inoculation.

Further research is also needed to eliminate persisting contaminants. Adjustments of the existing methods might be effective (e.g., repetition of treatments, exposure to treatments, and the concentration of antibiotics), but will not be able to overcome the potential problem of antibiotic resistance. Although not a solution, to get rid of contaminants will be their study. Persisting contaminants are commonly treated as disadvantageous, but they can also be seen as assets. They are very robust against treatments and might be interesting for industrial applications. In particular, alkaliphilic heterotrophic bacteria are in the focus of applied research, as they contain detergent enzymes which are operationally stable at elevated pH [61]. They have unique metabolic pathways which might be exploited in the future to treat wastewater, pollutants, dyes, and effluents of mining [62,63].

## 5. Conclusions

To obtain reliable results, it is mandatory to use appropriate media for testing axenicity. LB-agar, which is commonly used for screening axenicity in routine laboratories, turned out to be inappropriate for extremophile cultures. Instead, it is highly recommended to include a growth medium comparable to that of the target organism. In addition, a combination of cultivation-independent methods should be applied to validate axenicity.

Although we tried various treatments to achieve axenicity, we were not able to find an effective method. However, every failure has its advantage. By means of sequencing, we identified contaminants, which might be highly interesting for various purposes. Obviously, these taxa are robust, because they survived very aggressive treatments. Some of them might be promising for in-depth studies to commercially exploit them.

## Figures and Tables

**Table 1:** Summary of the axenic treatments. The differences from the standard treatment are highlighted in bold and underlined.

Standard Treatment (ST)	ST + Ultrasonication	ST + Chloramphenicol
Washing on filter	Washing on filter	Washing on filter
pH 12 for 72 h Antibiotics for 48 h Ampicillin (61.6 µg mL^−1^) Penicillin (85.8 µg mL^−1^) Cefoxitin (76.9 µg mL^−1^) Meropenem (38.9 µg mL^−1^)	pH 12 for 72 h **Ultrasonication** Antibiotics for 48 h Ampicillin (61.6 µg mL^−1^) Penicillin (85.8 µg mL^−1^) Cefoxitin (76.9 µg mL^−1^) Meropenem (38.9 µg mL^−1^)	pH 12 for 72 h Antibiotics for 48 h Ampicillin (61.6 µg mL^−1^) Penicillin (85.8 µg mL^−1^) Cefoxitin (76.9 µg mL^−1^) Meropenem (38.9 µg mL^−1^) **Chloramphenicol (10 µg mL^−1^) + glucose (100 µg mL^−1^)**

**Table 2:** Summary of plate screening. ST = standard treatment, ST + CHL = standard treatment + chloramphenicol, and ST + U = standard treatment + ultrasonication. LB did not show any bacterial lawns thus erroneously indicating axenicity (-).

	LB	R2A	Zarrouk
C	-	White lawn + red lawn after 1 week	A lot of white colonies + red lawn after some time
ST	-	White colonies after some weeks	Few white colonies, grew after some time
ST + CHL	-	After some weeks “paw-shaped” colonies	Few white colonies, grew after some time + big red colonies after 2 weeks
ST + U	-	After some weeks “paw-shaped” colonies + big red and round colonies	Few white colonies, grew after some time + big red colonies after 2 weeks

## Data Availability

The amplicon sequencing data generated in this study have been submitted to NCBI under the BioProject ID PRJNA866304.
